# Effect of Weakly Basic Conditions on the Separation and Purification of Flavonoids and Glycosides from *Chrysanthemum morifolium* Tea

**DOI:** 10.3390/molecules24020297

**Published:** 2019-01-15

**Authors:** Yuxiao Wang, Zhenzhen Xu, Yue Wu, Mo Li, Sicheng Pang, Zhe Liang, Yuanying Ni

**Affiliations:** 1 College of Food Science and Nutritional Engineering, China Agricultural University, Beijing 100083, China; wangg5211@163.com (Y.W.); wy1102742656@163.com (Y.W.); limo_0125@163.com (M.L.); 13031162533@163.com (S.P.); liangzhe@cau.edu.cn (Z.L.); 2 Institute of Quality Standard & Testing Technology for Agro-Products, Chinese Academy of Agricultural Sciences, Beijing 100083, China; xuzhenzhen@caas.cn; 3 National Engineering Research Center for Fruit and Vegetable Processing, Beijing 100083, China; 4 Key Laboratory of Fruit and Vegetable Processing, Ministry of Agriculture, Beijing 100083, China

**Keywords:** weakly basic condition, *Chrysanthemum morifolium* tea, chlorogenic acid, apigenin-7-*O*-glucoside, hydrolysis

## Abstract

Tea brewed from chrysanthemum flowers has been widely used in Chinese medicine. The possibly medicinal compounds in *Chrysanthemum morifolium* tea can be purified by preparative high performance liquid chromatography (HPLC), but this is usually done with acidic conditions, which leads to the hydrolysis of glycosides. In hopes of avoiding this hydrolysis, we explored the effect of weakly basic conditions on the separation and purification of flavonoids and glycosides from *Chrysanthemum morifolium*. We also explored the effects of weakly basic conditions on chlorogenic acid (3-CQA) and apigenin-7-*O*-glucoside (A7G). Our results show that the concentration of the weakly basic ammonium hydrogen carbonate and time had no significant effect on A7G, *p* < 0.01, but it had a significant effect on 3-CQA, *p* < 0.01. HPLC and ultraviolet (UV) analysis showed that the structure of 3-CQA is destroyed in weakly basic conditions. Caffeic acid, quinic acid, and 3,4-dihydroxymandelic acid, which is a hydrolysate of 3-CQA, were identified by ultra-performance liquid chromatography quadrupole time-of-flight mass spectrometry (UPLC-Q-TOF-MS). The results showed that weakly basic conditions could be used for the purification of flavonoids and glycosides but not for caffeoylquinic acids. Moreover, our work clarified the hydrolysis behaviour of caffeoylquinic acids, which can be helpful for research into their functional aspects.

## 1. Introduction

Tea brewed from chrysanthemum flowers has been extensively used for centuries to treat a variety of medical conditions, including chest pain, high blood pressure, diabetes, and headaches. The benefits of *Chrysanthemum morifolium* are closely related to the composition and content of phenolic compounds [1,2,3]. Many functional ingredients from *Chrysanthemum morifolium* tea have been investigated [3,4,5,6,7,8]. Guzelmeric [3] determined the fingerprint of apigenin-7-*O*-glucoside isolated from *Chrysanthemum morifolium* flowers and related species by high performance thin-layer chromatography (HPTLC). Uehara [4] isolated five flavonoid glycosides (luteolin-7-*O*-glucoside and 7-*O*-glucuronides of luteolin, apigenin, eriodictyol, and naringenin) from chrysanthemum species by HPLC with a photodiode array (PDA). Lin [5] identified 15 flavonoids and 15 caffeic acid derivatives isolated from *Chrysanthemum morifolium* by liquid chromatography with a diode array and characterised by electrospray ionisation/mass spectrometry (LC–DAD–ESI/MS). Han [6] identified apigenin-7-*O*-6′′-malonyl-glucoside, luteolin-7-*O*-rutinoside, quercetin-7-*O*-galactoside, quercetin-3-*O*-glucoside, apigenin-7-*O*-rutinoside, and chlorogenic acid from *Chrysanthemum morifolium* species by ultra-performance liquid chromatography quadrupole time-of-flight mass spectrometry (UPLC-Q-TOF-MS). Yuan [9] analysed chlorogenic acid, luteolin-7-*O*-β-d-glucoside, 3,5-di-caffeoylquinic acid, apigenin-7-β-d-glucopyranoside, and luteolin from chrysanthemum flowers by HPLC-DAD. These studies showed that chrysanthemum flowers contain caffeoylquinic acids, flavonoids, and glycosides.

Sensitive and efficient methods for the purification of these functional compounds from chrysanthemums are scarce in the scientific literature. The most common methods for separation and purification have been column chromatography, preparative high-performance liquid chromatography (Pre HPLC) [10], high-speed countercurrent chromatography (HSCCC) [11], and thin-layer chromatography (TLC) [12]. Unlike other methods, Pre-HPLC can isolate the many components of *Chrysanthemum morifolium* tea simultaneously, but the acid used for improving peak performance would hydrolyse the glycosides.

It is unknown whether weakly basic conditions will help to purify and analyse the components of *Chrysanthemum morifolium*, thus we explored this possibility by: (1) analysis of the components isolated in weakly basic conditions by HPLC-DAD; and (2) investigation of the hydrolytic behaviour of chlorogenic acid (3-CQA) and apigenin-7-*O*-glucoside (A7G) with UPLC-Q-TOF-MS. We used ammonium hydrogen carbonate to provide the weakly basic conditions because it decomposes above 36 °C to form ammonia, carbon dioxide, and water, which are easily removed from solvent systems. This study provides an effective approach for isolating flavonoids and glycosides from *Chrysanthemum morifolium* and clarifies the hydrolysis mechanism of caffeoylquinic acids, which will be helpful for research on the purification and analysis of related substances.

## 2. Results and Discussion

### 2.1. Analysing the Components of Chrysanthemum in Weakly Basic Conditions

**Figure 1:**
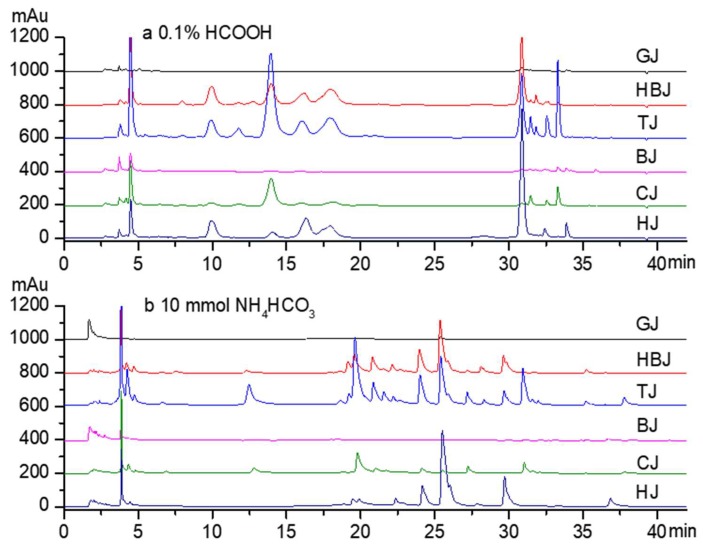
HPLC analysis of the components from *Chrysanthemum morifolium* tea for acidic and basic conditions. The gradient elution solvents consisted of phase A and phase B (acetonitrile). (**a**) Phase A was water with 0.1% formic acid, and (**b**) phase B was water with 10 mmol/L ammonium hydrogen carbonate. GJ, *C. morifolium* ‘Gongju’; HBJ, *C. morifolium* ‘Hangbaiju’; TJ, *C. morifolium* ‘Taiju’; BJ, *C. morifolium* ‘Boju’; CJ, *C. morifolium* ‘Chuju’; HJ, *C. morifolium* ‘Huangju’.

Figure 1a shows the components that can be isolated from *Chrysanthemum morifolium* in optimised acidic conditions. The components that were previously identified by Q-TOF-MS [10] mainly include chlorogenic acid (3-CQA), luteolin-7-*O*-glucoside, 3,5-dicaffeoylquinic acid, apigenin-7-*O*-glucoside (A7G), apigenin-7-*O*-glucuronide, luteolin-7-*O*-6′′-malonylglucoside, and apigenin. Figure 1a shows that these compounds are in lower concentrations in *C. morifolium* ‘Gongju’ and ‘Boju’ than for other cultivars. The concentrations of 3-CQA and 3,5-dicaffeoylquinic acid in *C. morifolium* ‘Taiju’ are higher than for other cultivars. There are much higher concentrations of A7G and apigenin in *C. morifolium* ‘Huangju’. The introduction of formic acid increased the retention and separation effect in the purification process by preparative HPLC, but in the acidic conditions, the glycosides hydrolysed [10].

We investigated the effect of weakly basic conditions on the separation of the components from chrysanthemum flowers (see Figure 1b). Most reversed phase C18 columns require acidic conditions rather than basic conditions, but they can be used in basic conditions with different packing materials or bonding methods. We used a C18 column (Athena C18-WP, 250 × 4.6 mm i.d., 5 μm, Anpel Laboratory Technologies Inc., Shanghai, China) that works in basic conditions. Because ammonium hydrogen carbonate (NH_4_HCO_3_) solutions are weakly basic, and because NH_4_HCO_3_ decomposes into the easily removed NH_3_, CO_2_, and H_2_O, we chose it to generate the weakly basic conditions. The gradient elution solvents that consisted of phase A (water with 10 mmol/L NH_4_HCO_3_) and phase B (acetonitrile) were optimised as follows: from 0 to 10 min with 10–10% B, from 10 to 20 min with 10–20% B, from 20 to 30 min with 20–30% B, and from 30 to 42 min with 30–42% B. We found that the components of the chrysanthemum flowers could also be separated with HPLC under weakly basic conditions.

### 2.2. Retention Rate of 3-CQA and A7G under Basic Conditions

**Figure 2:**
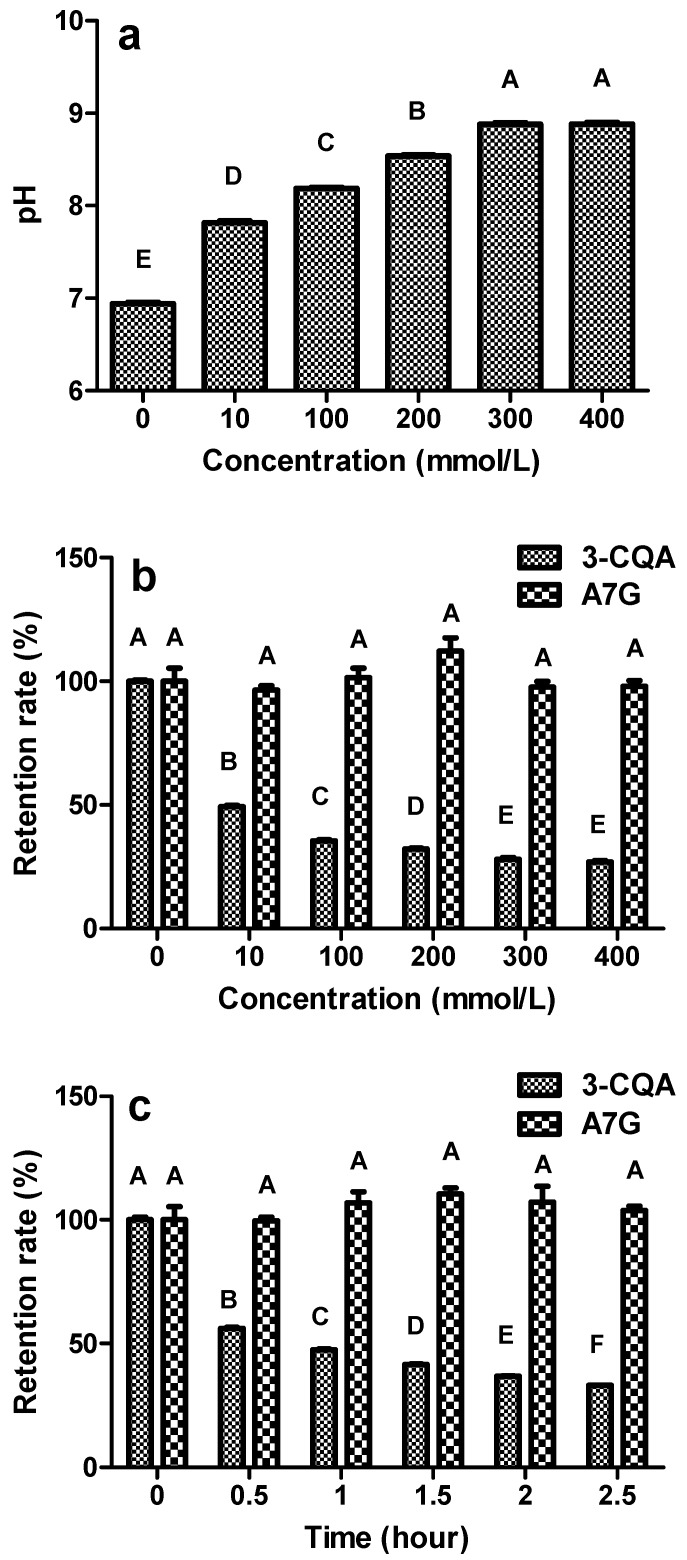
The effect of basic conditions on the retention rates of 3-CQA and A7G. Results using: (**a**) pH values of ammonium hydrogen carbonate, (**b**) various concentrations of ammonium hydrogen carbonate ranging from 0 to 400 mmol/L, and (**c**) time from 0 to 2.5 h. Means indicated by different letters differed significantly with a value of *p* < 0.01.

The 3-CQA and A7G were used as targets to investigate the effect of weakly basic conditions on the retention rate of caffeoylquinic acids and glycosides. Without ammonium hydrogen carbonate, the pH value of ultrapure water was 6.85 (Figure 2a). At concentrations of 300 and 400 mmol/L NH_4_HCO_3_, the pH value was below 9.0, which indicated that ammonium hydrogen carbonate is weakly basic.

The retention rates of 3-CQA decreased as the concentration of the ammonium hydrogen carbonate increased. Figure 2b shows that the retention rate of 3-CQA decreased to 49.2% when mixed with 10 mmol/L NH_4_HCO_3_ for 1 h. The retention rate of 3-CQA with 10 mmol/L NH_4_HCO_3_ was significantly lower than it was without the treatment with ammonium hydrogen carbonate, *p* < 0.01. The concentrations of ammonium hydrogen carbonate had no significant effect on the retention rates of A7G, *p* < 0.01. All of the retention rates for A7G were over 90% when mixed with different concentrations of ammonium hydrogen carbonate for 1 h. Unlike the acidic conditions, the weakly basic conditions did not lead to the hydrolysis of glycosides.

The retention rate of 3-CQA was significantly affected by time, *p* < 0.01. Figure 2c shows that the retention rates of 3-CQA mixed with 10 mmol/L NH_4_HCO_3_ decreased with increasing time. The time had no significant effect on the retention rates of A7G with 10 mmol/L NH_4_HCO_3_, *p* < 0.01. All the results showed that the weakly basic conditions could be used for separating flavonoids and glycosides but they are not be suitable for the purification of caffeoylquinic acids.

### 2.3. HPLC and UV Analysis

**Figure 3:**
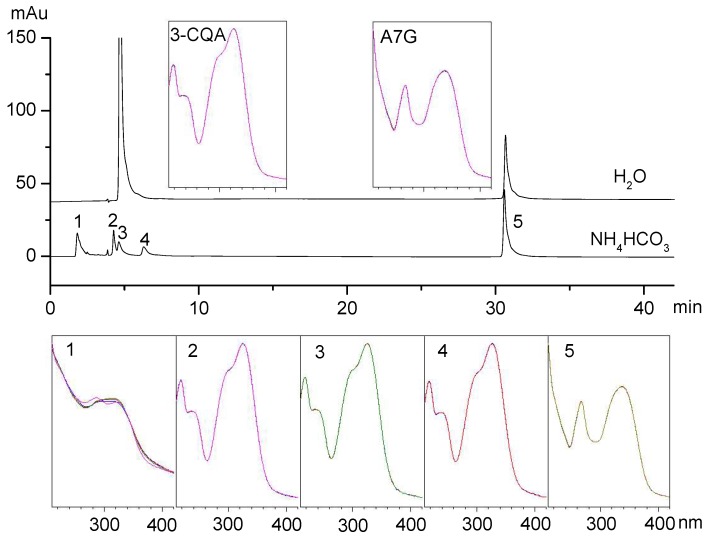
HPLC and UV analysis of the hydrolysates of 3-CQA and A7G treated with ammonium hydrogen carbonate. The UV spectrum is from a scan from 210 to 420 nm.

Ammonium acetate aqueous solutions have a pH of 7, so they could be used to eliminate the effect of acidic and basic conditions on 3-CQA and A7G. The gradient elution solvents consisted of phase A (water with 10 mmol/L NH_4_CH_3_CO_2_) and phase B (acetonitrile), using the same procedure as described for the weakly basic conditions. Without pretreatment with ammonium hydrogen carbonate, there was only one peak for 3-CQA and one peak for A7G in HPLC, as seen in Figure 3. With pretreatment with ammonium hydrogen carbonate, peak 5 in Figure 3 is consistent with the retention time and ultraviolet spectrum of A7G, indicating that A7G was stable in weakly basic conditions, but there were three new peaks generated (peaks 1, 2, and 4 in Figure 3).

The new peaks 1 and 4 in the UV spectrum were consistent with structure of 3-CQA, while the retention time was different, indicating that they were the isomers of 3-CQA. The 1-CQA, 4-CQA, and 5-CQA were the major 3-CQA isomers [13,14,15]. The retention time and UV spectra of peak 3 was consistent with 3-CQA, but it is unclear whether this was due to the remaining 3-CQA or an isomer.

Farah [16] studied the effect of roasting on the formation of chlorogenic acid lactones in coffee and found that roasting causes the isomerisation of chlorogenic acids prior to the formation of lactones, and that the levels of lactones in roasted coffee do not reflect the levels of precursors in green coffee. Xie [17] investigated the isomeric transformations of chlorogenic acid in buffers by UPLC-Q-TOF-MS and proved that in a phosphate buffer (pH 7.4), *trans*-5-CQA first isomerises to *trans*-4-CQA and then to *trans*-3-CQA by intramolecular acyl migration. When exposed to UV light, *trans*-3, -4, and -5-CQA undergo *cis*/*trans* isomerisation to form cis isomers. The isomerisation was dependent on the pH and the incubation temperature.

### 2.4. UPLC-Q-TOF-MS Analysis

**Figure 4:**
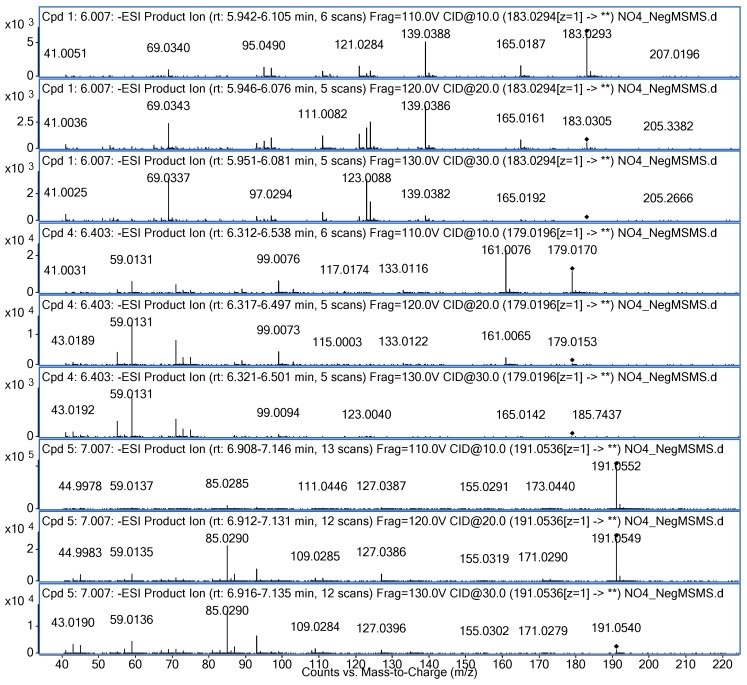
Target MS/MS analysis by UPLC-Q-TOF-MS of the hydrolysates of 3-CQA treated with ammonium hydrogen carbonate. MS/MS data for 3-CQA was obtained in negative ESI mode. MS/MS data was for collision energies of 10 eV, 20 eV, and 30 eV. Cpd represents compound. Compounds **1**, **4** and **5** were identified as 3,4-dihydroxymandelic acid, caffeic acid, and quinic acid, respectively.

The hydrolysis behaviour of 3-CQA was further investigated by UPLC-Q-TOF-MS. The formula and mass of hydrolysates were calculated using the MassHunter Workstation software (2014, version B.07.00, Agilent Technologies, Inc., Santa Clara, CA, USA). Figure 4 shows that the three hydrolysates had (1) a formula C_8_H_8_O_5_ and a mass of 183.0294 [M − H]^−^, (2) a formula C_5_H_8_O_7_ and a mass of 179.0196 [M − H]^−^, and (3) a formula C_7_H_12_O_6_ and a mass of 191.0536 [M − H]^−^. The compound with the formula C_5_H_8_O_7_ had product ion *m*/*z* ratios of 161.0076, 99.0076, and 59.0131. The compound with the formula C_7_H_12_O_6_ had product ion *m*/*z* ratios of 85.0290, 93.0340, and 127.0386. The fact that these results had a highly matching score with the results of a search of the METLIN metabolite database (supported by Agilent Technologies) led to the conclusion that the formulas C_5_H_8_O_7_ and C_7_H_12_O_6_ represented caffeic acid and quinic acid.

The compound of formula C_8_H_8_O_5_ had a major ion *m*/*z* ratio of 183.0294 [M − H]^−^ and product ion *m*/*z* ratios of 165.0187, 139.0388, 123.0088, and 69.0337. The previous literature [13,14,15,16,17] focused on the isomers of 3-CQA, while there was no literature about this hydrolysate of 3-CQA (peak 1 in Figure 3). We first used human metabolome databases (HMDB, http://www.hmdb.ca/) and Scifinder (https://scifinder.cas.org/) to search for this hydrolysate of 3-CQA. A comparison with the MS and MS/MS data led to the conclusion that this hydrolysate (peak 1 in Figure 3) was 3,4-dihydroxymandelic acid.

**Figure 5:**
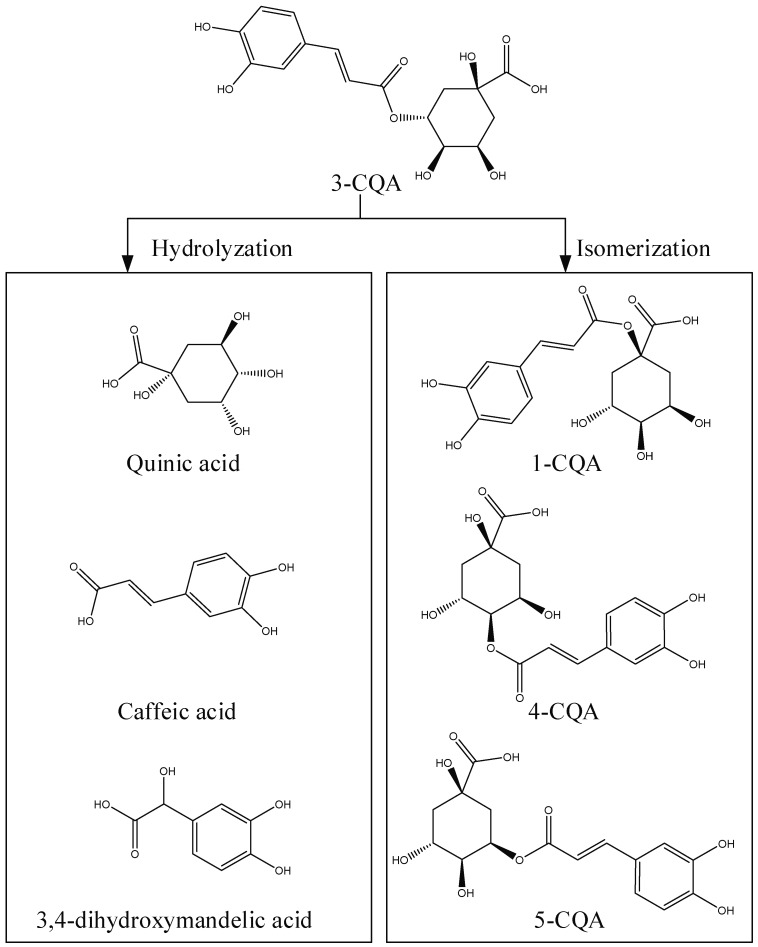
The proposed hydrolysation and isomerisation of 3-CQA in weakly basic conditions. 1-CQA represents 1-caffeoylquinic acid, 3-CQA represents chlorogenic acid, 4-CQA represents 4-caffeoylquinic acid, and 5-CQA represents 5-caffeoylquinic acid.

Figure 5 shows the hydrolysis pathways of 3-CQA. There were two hydrolysis pathways of chlorogenic acid in basic conditions, hydrolysation and isomerisation. Besides the isomeric transformations, the hydrolysates of 3-CQA are caffeic acid, quinic acid, and 3,4-dihydroxymandelic acid.

## 3. Materials and Methods

### 3.1. Materials

Six kinds of *Chrysanthemum morifolium* cultivars were purchased from different companies (Table 1). Apigenin-7-*O*-glucoside, chlorogenic acid, formic acid, acetonitrile, ammonium hydrogen carbonate, and ammonium acetate were obtained from Sigma-Aldrich (St Louis, MO, USA). Ultrapure water (18.2 MΩ cm) was further purified using a Milli-Q system (Millipore, Billerica, MA, USA). Unless otherwise mentioned, the chemicals and reagents used in this experiment were of analytical grade.

### 3.2. Sample Preparation

In this study, the flowers of *C. morifolium* ‘Gongju’, *C. morifolium* ‘Hangbaiju’, *C. morifolium* ‘Tai’, *C. morifolium* ‘Boju’, *C. morifolium* ‘Chuju’ and *C. morifolium* ‘huangju’ were ground to a powder and screened through a 100-mesh filter. The samples were stored at −80 °C before analysis.

### 3.3. Extraction with Ultrasound Method

A previously reported ultrasound method [10] was used to extract the components of the chrysanthemums. Initially, 0.1000 ± 0.0005 g of chrysanthemum powder was added to a 2 mL polyethylene tube, and 1 mL of ultrapure water was added. After being vortexed, the tube was placed in an ultrasonic cleaner for rapid extraction. The ultrasound conditions were set as follows: an extraction time of 35 min, a temperature of 50 °C, and an ultrasound power of 350 W. Then the solution was centrifuged with a high-speed refrigerated centrifuge (CR 21G III, Hitachi, Chiyoda-Ku, Japan) at 10,000 rpm for 1 min. The resulting extracts were filtered through a 0.22 μm nylon membrane before analysis.

### 3.4. Separation of the Components under Weakly Basic Conditions by HPLC

Analytical HPLC was performed using an Agilent 1260 HPLC equipped with a degasser, quad pump, autosampler, and diode array detector (DAD). Samples were scanned (210–420 nm) with stored wavelengths of 327 nm and 254 nm. In acidic conditions, the components were analysed according to a previously described method [10] with a Venusil ASB C18 column (250 × 4.6 mm i.d., 5 μm, Agela Technologies, Tianjin, China) with a flow rate of 1 mL/min, an injection volume of 10 μL, and a temperature of 30 °C. The gradient elution solvents composed of phase A (water with 0.1% formic acid) and phase B (acetonitrile) were as follows: 0–25 min with 20–20% B, 25–30 min with 20–50% B, 30–35 min with 50–100% B, 35–37 min with 100–20% B, and 37–42 min with 20% B.

In weakly basic conditions, the gradient elution solvents consisted of phase A (water with 10 mmol/L ammonium hydrogen carbonate) and phase B (acetonitrile). The analytical conditions were optimised as follows: 0–10 min with 10–10% B, 10–20 min with 10–20% B, 20–30 min with 20–30% B, and 30–42 min with 30–42% B. Considering the tolerance of the column, another column (Athena C18-WP, 250 × 4.6 mm i.d., 5 μm, Anpel Laboratory Technologies Inc., Shanghai, China) was used to analyse the components under basic conditions.

### 3.5. Hydrolysis Behaviour of 3-CQA and A7G under Basic Conditions.

The hydrolysis behaviour of 3-CQA and A7G were investigated under different conditions (time and content of ammonium hydrogen carbonate). First, 10 μL 3-CQA solution (0.125 mg/mL) and 10 μL A7G solution (0.25 mg/mL) were mixed with 180 μL NH_4_HCO_3_ with concentrations ranging from 10 to 400 mmol/L, and the solutions were kept in a water bath at 50 °C for different lengths of time (0.5 to 2.5 h). A UPLC column (ACQUITY UPLC BEH C18, 2.1 × 100 mm i.d., 1.7 μm, Waters, Dublin, Ireland) was used to shorten the analysis time. Because an aqueous ammonium acetate solution is nearly neutral, it was used to eliminate the possible effects of acidic and basic conditions on 3-CQA and A7G. The two solvent phases were phase A (water with 10 mmol/L CH_3_COO NH_4_) and phase B (acetonitrile), and the analysis conditions were 0.1 mL/min with an injection volume of 5 μL and an isocratic elution with phase A and phase B (75:25, *v*/*v*). The retention rates of 3-CQA and A7G were calculated with and without ammonium hydrogen carbonate using the following equation:

Retention rate (%)=A1/A0×100

where *A1* represents the peak area calculated for mixed with different concentrations of ammonium hydrogen carbonate, and *A0* represents the peak area without the treatment of ammonium hydrogen carbonate.

### 3.6. Analysis of the Hydrolysates of 3-CQA and A7G with HPLC and UV

The hydrolysates of 3-CQA and A7G were analysed with HPLC. The two-phase solvents consisted of phase A (water with 10 mmol/L CH_3_COONH_4_) and phase B (acetonitrile), and a column (Athena C18-WP, 250 × 4.6 mm i.d., 5 μm, Anpel Laboratory Technologies Inc., Shanghai, China) was used to analyse the hydrolysates. The gradient elution was the same as for the weakly basic conditions, which were described in Section 3.4. The UV spectrum was determined with a diode array detector (DAD) that scanned from 210 to 420 nm.

### 3.7. UPLC-Q-TOF-MS Analysis

The hydrolysates were further analysed with a UPLC-Q-TOF-MS system (1290 Infinity II, G6530C Q-TOF, Agilent). The UPLC analysis was done with a UPLC column (ACQUITY UPLC BEH C18, 2.1 × 100 mm i.d., 1.7 μm, Waters, Ireland), a phase A of water with 10 mmol/L CH_3_COO NH_4_, a phase B of acetonitrile, and an isocratic elution with phase A and phase B (95:5, *v*/*v*). The MS conditions were a gas temperature of 250 °C, a drying gas flow of 7 L/min, a sheath gas temperature of 350 °C, a sheath gas flow of 11 L/min, a capillary voltage of 3500 V, a nozzle voltage of 0 V, a fragmentor voltage of 130 V, and a skimmer voltage of 65 V. The MS range was 100–1700 *m*/*z* with an acquisition rate of 4 spectra/s and an acquisition time of 250 ms/spectrum. The MS/MS range was 100–1000 *m*/*z* with an acquisition rate of 3 spectra/s and an acquisition time of 333.3 ms/spectrum. The targeted MS/MS fixed collision energies were 10 eV, 20 eV, and 30 eV. The MS data was qualitatively analysed using MassHunter Workstation software (version B.07.00, coupled with the METLIN metabolite database supported by Agilent Technologies.

## 4. Conclusions

Due to the problem of the hydrolysis of glycosides when separating the components of *Chrysanthemum morifolium* in acidic conditions, weakly basic conditions were investigated for this separation. Ammonium hydrogen carbonate aqueous solutions are weakly basic, and because NH_4_HCO_3_ decomposes to form easily-separated NH_3_, CO_2_, and H_2_O, NH_4_HCO_3_ solutions were used as the HPLC solvent. HPLC analysis showed that the components from *Chrysanthemum morifolium* could be separated with weakly basic conditions. As with the purification with acidic solutions, weakly basic conditions could hydrolyse caffeoylquinic acids, but they have no effect on flavonoids and glycosides. There were two hydrolysis pathways of chlorogenic acid in basic conditions: hydrolysation and isomerisation. The 3-CQA could be isomerised to 1-CQA, 4-CQA, and 5-CQA, and caffeic acid, quinic acid, and 3,4-dihydroxymandelic acid have been proposed as hydrolysates of 3-CQA. The results showed that weakly basic conditions could be used for the purification of flavonoids and glycosides, but not for caffeoylquinic acids. This study not only provided a procedure for the separation and purification of flavonoids and glycosides from *Chrysanthemum morifolium* in weakly basic conditions, it also clarified the hydrolysis behaviour of caffeoylquinic acids, which could be helpful for further investigations into their functional aspects.

## Figures and Tables

**Table 1:** Samples and origins of the studied *Chrysanthemum morifolium* flower tea from China.

Samples	Codes	Company	Origins
*C. morifolium* ‘Gongju’	GJ	Beijing Tongrentang Health Pharmaceutical Industry Co., Ltd.	Huangshan City, Anhui Province
*C. morifolium* ‘Hangbaiju’	HBJ	Beijing Tongrentang Health Pharmaceutical Industry Co., Ltd.	Tongxiang City, Zhejiang Province
*C. morifolium* ‘Taiju’	TJ	Beijing Zhang Yiyuan Jinqiao Tea Co., Ltd.	Zhongwei City, Ningxia Hui Autonomous Region
*C. morifolium* ‘Boju’	BJ	Bozhou Zhongyitang Chinese Medicinal Materials Sales Co., Ltd.	Bozhou City, Anhui Province
*C. morifolium* ‘Chuju’	CJ	Anhui Jutai Chuju Herb Science and Technology Co., Ltd.	Chuzhou City, Anhui Province
*C. morifolium* ‘Huangju’	HJ	Huangshan Dingxiangwu Ecological Agriculture Development Co., Ltd.	Shangrao City, Jiangxi Province
